# Selective accumulation of ALA-induced PpIX and photodynamic effect in chemically induced hepatocellular carcinoma

**DOI:** 10.1038/sj.bjc.6601135

**Published:** 2003-08-12

**Authors:** M Otake, M Nishiwaki, Y Kobayashi, S Baba, E Kohno, T Kawasaki, Y Fujise, H Nakamura

**Affiliations:** 1 Second Department of Internal Medicine, Hamamatsu University School of Medicine, Handayama 1-20-1, Hamamatsu, Shizuoka 431-3192, Japan; 2 Second Department of Pathology, Hamamatsu University School of Medicine, Handayama 1-20-1, Hamamatsu, Shizuoka 431-3192, Japan; 3 Photon Medical Research Center, Hamamatsu University School of Medicine, Handayama 1-20-1, Hamamatsu, Shizuoka 431-3192, Japan; 4 Department of Chemistry, Hamamatsu University School of Medicine, Handayama 1-20-1, Hamamatsu, Shizuoka 431-3192, Japan

**Keywords:** photodynamic therapy (PDT), photodynamic diagnosis, hepatocellular carcinoma, chemically induced hepatocellular carcinoma, 5-aminolaevulinic acid, excimer dye laser

## Abstract

The possibility of 5-aminolaevulinic acid-based photodynamic therapy (ALA-PDT) for liver cancer was investigated using a chemically induced hepatocellular carcinoma (HCC) model. Endogenously synthesised protoporphyrin IX (PpIX) following the administration of ALA is an effective photosensitiser for PDT. We determined the fluorescence intensity of PpIX in HCC and nontumoral tissue in the liver. 5-Aminolaevulinic acid was intravenously injected to male Fisher rats with HCC at a dose of 500 mg kg^−1^, and the fluorescence intensity in each tissue sample excised from liver was measured with a spectrofluorometer at 1, 3 and 6 h after administration. Fluorescence intensity was at a peak of 3 h after administration in both HCC and nontumoral tissue. The accumulation of PpIX in HCC was higher than that in the nontumoral tissue at 1 h (*P*<0.001) and 3 h (*P*<0.05) after ALA administration. Based on these results, PDT was performed on HCC at 3 h after 500 mg kg^−1^ ALA administration before laser irradiation of 30 J per tumour. Antitumour effect was more evident in HCC than in the nontumoral tissue surrounding HCC. These findings suggest the possibility to detect HCC by fluorescence and to treat HCC by light.

Hepatocellular carcinoma (HCC) is considered to be a highly therapy-resistant malignancy. Although various kinds of palliative treatment strategies, such as transcatheter arterial embolisation (TAE), percutaneous ethanol injection therapy (PEIT) and percutaneous microwave coagulation therapy (PMCT), have been employed clinically, the prognosis remains unsatisfactory. New strategies are required to cure patients with HCC.

Photodynamic therapy (PDT) is a promising local treatment modality based on the selective accumulation of a photosensitiser in malignant tissue and the subsequent illumination with laser light of an appropriate wavelength. In recent years, this therapy has been successfully used in the management of patients with various malignant tumours, including cancers of the skin (Fritsch *et al*, 1998), gastrointestinal tract (Mimura *et al*, 1996), lung (Kato *et al*, 1989) and uterus (Muroya *et al*, 1996). Photodynamic therapy has been considered an unsuitable treatment modality for HCC, because of the poor tumour-selective accumulation of conventional photosensitiser compared with nontumoral tissue (Gomer and Dougherty, 1979; Bugelski *et al*, 1981; Bellnier *et al*, 1989). Recently, substantial research has been invested in finding new tumour localisers with more optimal characteristics for PDT against HCC.

5-Aminolaevulinic acid (ALA) is a natural precursor to protoporphyrin IX (PpIX) in the haem biosynthesis pathway of biological systems. In normal cells, the synthesis of haem and the precursor PpIX is regulated by a feedback control system. It has been suggested that this feedback system can be set out of control if a high amount of ALA is applied to the system exogenously. The investigators have found that exogenous administration of ALA can induce the cellular PpIX accumulation (Malik and Lugaci, 1987; Hua *et al*, 1995), and this PpIX can be an efficient photosensitiser (Kennedy and Pottier, 1992). Moreover, it has been reported that the accumulation of PpIX is greater in malignant than in normal tissues because of the alteration in the enzyme profile of the haem biosynthesis pathway (Abels *et al*, 1994; Kriegmeir *et al*, 1996).

Egger *et al* (1997) indicated that porphyrin accumulation after ALA administration was greater and continued to increase for a longer period of time in hepatoma cells than in normal hepatocytes. Moreover, Svanberg *et al* (1996) reported that PDT for transplanted colon cancer to the normal liver was effective. These results suggest that ALA may be useful for PDT of HCC. However, the HCC models used in the previous papers did not reflect the circumstances of human HCC. The present study is the first to report on PDT in chemically induced HCC, a model of HCC *in situ.* Differences between cancerous and noncancerous tissue and haemodynamics reflect human liver with HCC better than the previous hepatoma models. To establish whether PDT might be applicable to HCC, we investigated the possibility of selective accumulation of PpIX and PDT effect after administration of ALA to the chemically induced HCC model.

## MATERIALS AND METHODS

### Animals and tumour model

Male Fisher-344 rats (Japan SLC, Hamamatsu, Japan) aged 5 weeks weighing about 80 g were used for hepatocarcinogenesis. They were housed in animal quarters on a 12-h light–dark illumination schedule. They had free access to a standard pellet diet (Nippon Clea Co., Tokyo, Japan) and water. The rats were given distilled water containing 100 ppm of diethylnitrosamine (Tokyo Chemical Industrial Co., Tokyo, Japan) for 11 weeks to induce HCC, followed by tap water for 2 weeks. These chemically induced HCC model rats weighing approximately 200 g were used for this study.

In this experimental hepatocarcinogenesis model, the whole liver weighed 14.8 g ±3.2 (s.d.). In the liver, various sizes of tumour from 5 to 15 mm in diameter were observed. There were individual differences in tumour size and number among the animals. There were different types of hepatocellular lesions in this chemically induced HCC model at the time of using in this study. Referring to a report by Williams (1980) and the General Rules for the Clinical and Pathological Study of Primary Liver Cancer (Liver Cancer Study Group of Japan, 2000), the liver lesions were classified pathologically as follows: foci of altered hepatocytes (FAH, small sublobular lesions of hepatocytes that do not disrupt the parenchymal architecture), hyperplastic nodules (HN, hepatocytic lesions larger than the area of a lobule that disrupt and compress the surrounding parenchyma but that exhibit minimal to mild dysplasia), atypical nodules (AN, hepatocytic lesions larger than the area of a lobule that disrupt and compress the surrounding parenchyma and that exhibit moderate to severe dysplasia), HCC (hepatocytic lesions that exhibit advanced cellular and/or structural atypia consistent with criteria for human HCC). There was little normal liver tissue in this model. Nontumoral tissue mainly consisted of FAH and HN, and tumoral tissue mainly consisted of AN and HCC. This model differs from human HCC in that it is not associated with cirrhosis. However, the model showed many hyperplastic nodules resembling cirrhotic pseudolobules and formation of HCC and precancerous lesions, as in human cirrhosis.

All animal studies were carried out with the approval of our institutional ethical committee for the care and use of laboratory animals and met the standards required by the UKCCCR guidelines (Workman *et al*, 1998).

### Administration of ALA

Solutions of ALA (Cosmo Bio KK, Tokyo, Japan) were freshly prepared by dissolving 100 mg of ALA in 1 ml of phosphate-buffered saline (PBS: 136.9 mM sodium chloride, 2.7 mM potassium chloride, 8.1 mM disodium hydrogen phosphate, 1.5 mM potassium dihydrogen phosphate). The pH of the solution was adjusted to 5.0–6.5 using pH indicator paper (Whatman International Ltd, Maidstone, England) by addition of 1 N sodium hydride. This solution was used within 10 min after preparation. A total of 500 mg kg^−1^ of ALA was injected through the tail vein under ether anaesthesia.

The chemically induced HCC model rats were used to measure the fluorescence intensity at various time intervals after ALA administration. They were divided into four groups: group 1 (five rats), measured at 1 h after ALA administration; group 2 (five rats), measured at 3 h after ALA administration; group 3 (four rats), measured at 6 h after ALA administration; group 4 (four rats), measured without ALA administration.

### Fluorescence detection of PpIX in tissue homogenates

The tissue accumulation of ALA-induced porphyrins was determined by measuring the fluorescence of tissue homogenates at different times after intravenous administration of ALA. Autopsy was performed following the animals' death under deep anaesthesia, and the whole liver was removed. Tissue samples, excised from tumoral and nontumoral tissue of the liver, were frozen at −80°C until fluorescence measurements. The fluorescence measurements of the tumoral parts were performed for all tumours that were large enough to be measured. In each rat, fluorescence measurements of the nontumoral tissue were taken from at least three different sites. Some part of each tissue sample was used for histopathological study. The rest of the sample, mean 65.8 mg, was homogenised thoroughly in distilled water (100 mg wet tissue 6 ml^−1^ distilled water), and centrifuged at 3000 r.p.m. (1000**g**) for 5 min. The supernatants were transferred to a quartz cuvette and the cuvette was positioned in a spectrofluorometer (FP-777, Japan Spectroscopic Co. Ltd., Tokyo, Japan). The supernatants were excited at 410 nm, and fluorescence emission was scanned from 550 to 700 nm. Background signals were subtracted from the spectra of the supernatants without ALA. Maximum fluorescence intensity was detected at 635 nm, and this peak was selected for measurement of the porphyrin content in tissues. The settings of the fluorometer were adjusted to obtain the optimum signal in the least amount of time to avoid any possible photobleaching of the porphyrin.

**Figure 1:**
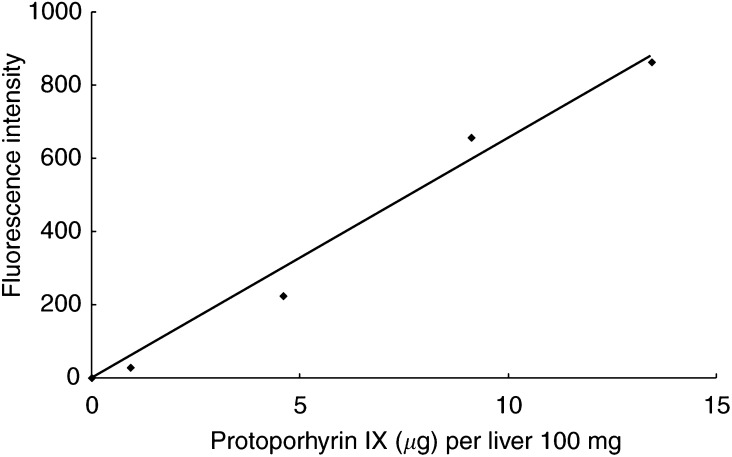
Standard curve made by the addition of known levels of PpIX Na to liver extracts from nontumoral parts without ALA administration. PpIX concentration is expressed as micrograms per liver 100 mg.

In the preliminary study, a standard curve was made by the addition of known levels of PpIX Na to liver tissue extracts from nontumoral tissue without ALA administration. The standard curve is shown in Figure 1

. It shows a near-linear relationship between fluorescence intensity measured by FP-777 and PpIX concentrations.

### Fluorescence imaging

Fluorescence imaging in sections of HCC and nontumoral tissue was achieved using fluorescence photography and microscopy in order to investigate the difference between HCC and nontumoral tissues. The whole liver was excised after the animals' death under deep anaesthesia at 3 h after intravenous administration of 500 mg kg^−1^ ALA. Fluorescence photography was performed using a 360 nm flash lamp, a colour camera (Bronica ETRSi, Tamron Co. Ltd., Japan) and lens (Zenzanon PE, Tamron Co. Ltd., Japan) (Onizawa *et al*, 1999). To characterise the autofluorescence elicited, an SC-52 Fuji filter (Fuji Film, Tokyo, Japan), in which fluorescence below 520 nm was absorbed, was set in front of the lens of the camera. Instant colour film FP-100C (Fuji Film, Tokyo, Japan) was used in the instant photography camera.

Next, we performed fluorescence micrography using the frozen sections of 10 *μ*m thickness cut by freezing microtome (Cryostats, Bright Instrument Co. Ltd, England). Tissue sections were prepared using a ProLong Antifade Kit (Molecular Probes, OR, USA) and imaged with a minimum of light exposure to avoid bleaching of porphyrin. A fluorescence microscope (Nikon TE300, Tokyo, Japan) and × 40 objective lens (NA=0.95) were used. Using an excitation filter (470±15 nm) and emission filter (BA 515 nm) (Nikon B2A, Tokyo, Japan), the specimen including HCC and nontumoral tissue was excited with blue light and epifluorescence above 520 nm was captured with negative film Superia 400 (Fuji Film, Tokyo, Japan).

### Photodynamic therapy

PDT was performed on HCC 5–9 mm in diameter (four tumours). ALA at a dose of 500 mg kg^−1^ was administered intravenously 3 h before laser irradiation. Excimer dye laser (Hamamatsu Photonics KK, Hamamatsu, Japan) was used to generate light at a wavelength of 630 nm. The output energy was 4 mJ per pulse, the repetition rate was 40 Hz, and the power density was 160 mW. An 800 *μ*m bare fibre was set to irradiate just above the HCC at a distance of 3–4 mm from the tumour surface. The duration of light exposure was 187 s for a total light dose of 30 J per tumour (47–90 J cm^−2^). Animals were killed 24 h after PDT, and PDT effects were evaluated histopathologically.

### Histopathological study

Some part of the tissue samples fixed in 20% formaldehyde solution were dehydrated and embedded in paraffin. Sections (3 *μ*m thick) cut from each paraffin-embedded specimen were deparaffinised and stained with haematoxylin and eosin (HE). All the sections were examined by the same pathologist and classified into four tissue types; HCC, AN, HN and FAH, and subsequently the fluorescence values of all samples were compared. Atypical nodules were excluded from the present study due to the insufficient number of measured sites for statistical investigation.

### Statistical analysis

For statistical analysis, Student's *t*-test was used to obtain a comparison of the fluorescence intensity between HCC and nontumoral tissue. The *P*<0.05 was considered to indicate a significant difference. The HCC/nontumoral tissue ratio was expressed as 95% confidence interval (CI).

## RESULTS

### Fluorescence intensity

**Figure 2:**
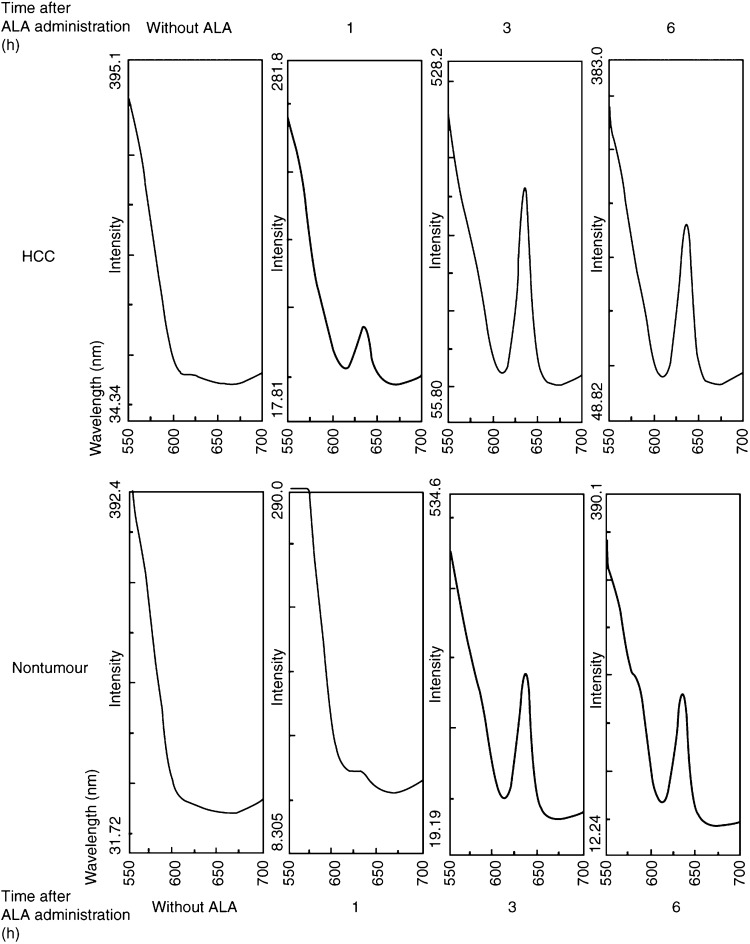
Representative spectra of HCC and nontumoral tissue at various times after ALA administration. The supernatants of tissue samples were excited at 410 nm, and fluorescence emission was scanned from 550 to 700 nm. Maximum fluorescence intensity was detected at 635 nm.

Maximum fluorescence intensity of the tissue homogenate was detected at 635 nm both in HCC and in nontumoral tissue after ALA administration. This peak was selected for measurement of the PpIX content in tissues because the peak was not detected in the tissue homogenate without ALA (Figure 2

).

### Analysis of tissue concentrations of PpIX

**Figure 3:**
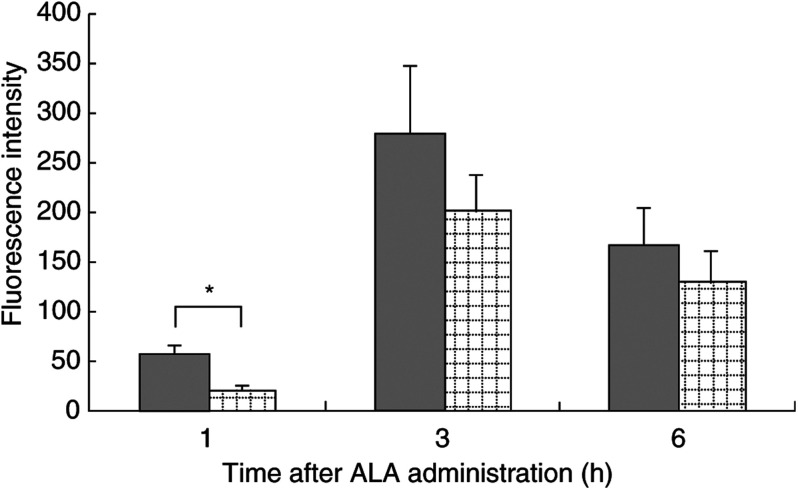
Fluorescence intensity of PpIX in HCC and in nontumoral tissue at various time intervals after intravenous administration of 500 mg kg^−1^ ALA. Bars (s.e.) are based on between eight and 19 tissues. ▪ HCC, 
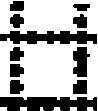
 nontumoral tissue. Significant difference: ^*^P<0.01.

Fluorescence intensity of PpIX in HCC and in nontumoral tissue was compared. The measurements are 16 HCCs and 15 nontumoral tissues at 1 h, 10 HCCs and 19 nontumoral tissues at 3 h, eight HCCs and 13 nontumoral tissues at 6 h. Fluorescence intensity in HCC and nontumoral tissue at various time intervals after 500 mg kg^−1^ ALA accumulation is shown in Figure 3

. The value was 56.6±9.7 (mean±s.e.) in HCC, 19.5±3.6 in nontumoral tissue at 1 h, 279.3±67.1 in HCC, 202.0±35.3 in nontumoral tissue at 3 h and 166.6±37.7 in HCC, 129.9±32.3 in nontumoral tissue at 6 h. The fluorescence intensity of PpIX is higher at 3 h than at 1 h and 6 h both in HCC and in the nontumoral tissue. Moreover, at any time, the fluorescence intensity of PpIX in HCC tends to be higher than that in nontumoral tissue, and the fluorescence intensity in HCC is significantly higher (*P*<0.01) than that in nontumoral tissues only at 1 h.

**Figure 4:**
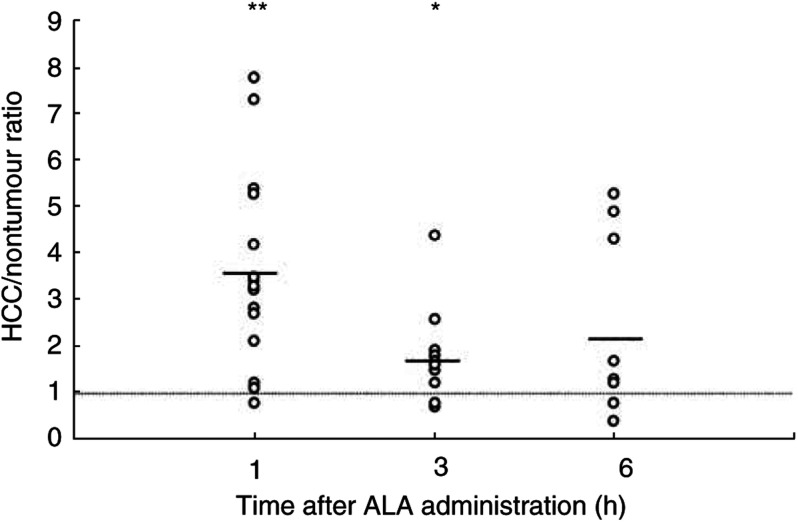
Ratio based on comparison of HCC to the nontumoral tissue of the same liver. Each dot indicates the ratio of the fluorescence intensity in the HCC to that in the nontumoral tissue (mean of three to six different sites). The threshold value at 1 is marked. The mean (bar) of this ratio at various times is 3.5 at 1 h, 1.8 at 3 h and 2.2 at 6 h after ALA administration. A 95% CI of the ratio is from 2.3 to 4.7 at 1 h, from 1.1 to 2.6 at 3 h and from 0.4 to 4.1 at 6 h. Significant difference: ^*^P<0.05, ^**^P<0.001.

Figure 3 shows the means of all measured values without taking individual differences into consideration. However, differences in PpIX accumulation in different tissue types in the same liver are important. The HCC/nontumoral tissue ratio was calculated for each individual liver. The denominator was the mean of measurements of at least three different sites of nontumoral tissue in each liver. The HCC/nontumoral tissue fluorescence intensity ratio is shown in Figure 4

. The mean of this ratio was greater than 1 at every time, which means that the concentration of PpIX in HCC is higher than in nontumoral tissue. Statistically, selective accumulation of PpIX in HCC was found at 1 (*P*<0.001) and 3 (*P*<0.05) hours after ALA administration.

### Fluorescence imaging

**Figure 5:**
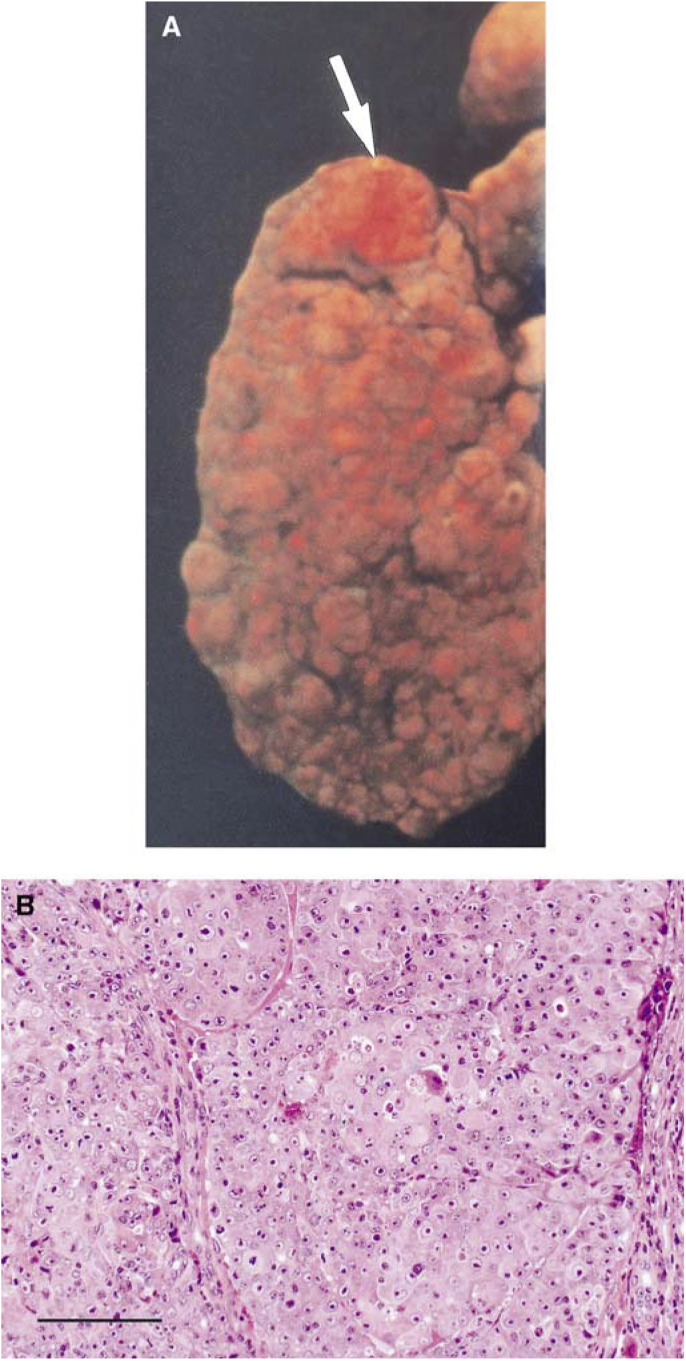
(**A**) Fluorescence photograph of the liver including HCC at 3 h after 500 mg kg^−1^ ALA administration. The tumour at the edge of the liver (arrow) was illuminated by excitation of blue light. (**B**) The specimen stained with HE. The tumour was revealed to be HCC. Scale: a bar represents 0.1 mm.

A fluorescence photograph of the liver at 3 h after 500 mg kg^−1^ ALA administration is shown in Figure 5

**Figure 6:**
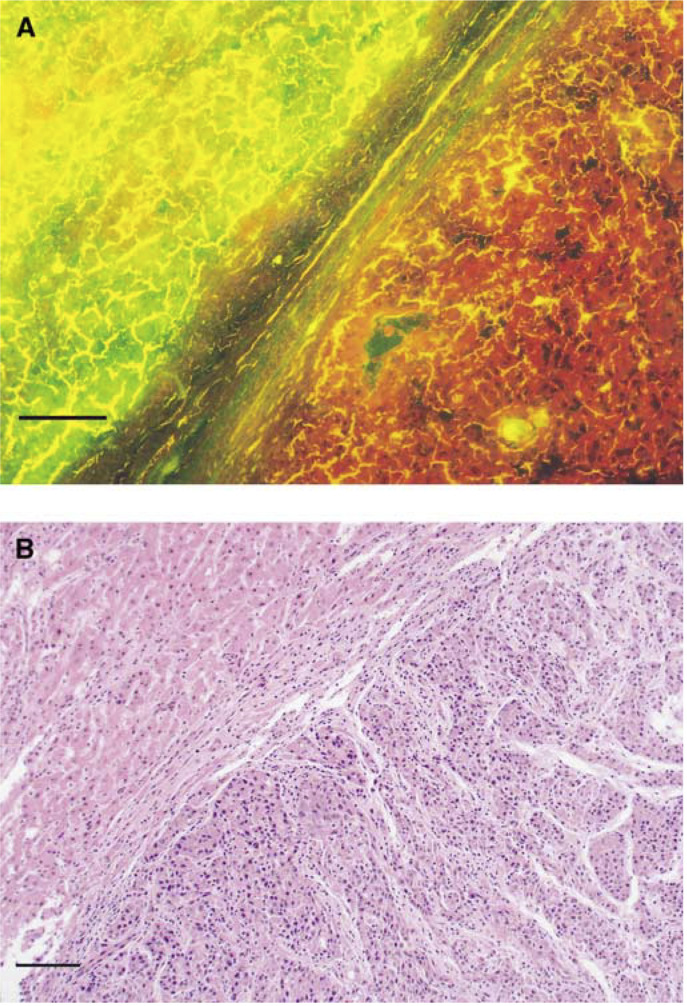
(**A**) Fluorescence micrograph of the liver including HCC at 3 h after 500 mg kg^−1^ ALA administration. The right part of the specimen was illuminated reddish-orange by excitation of blue light. (**B**) The specimen stained with HE. Histopathological examination revealed the right part to be HCC and the left part to be non-HCC. Scale: a bar represents 0.1 mm.

. HCC was illuminated by excitation of blue light. A fluorescence micrograph of the liver at 3 h after 500 mg kg^−1^ ALA administration is shown in Figure 6

. The specimen was illuminated entirely, with the HCC part illuminated reddish-orange homogeneously due to PpIX.

### Photodynamic therapy effect

**Figure 7:**
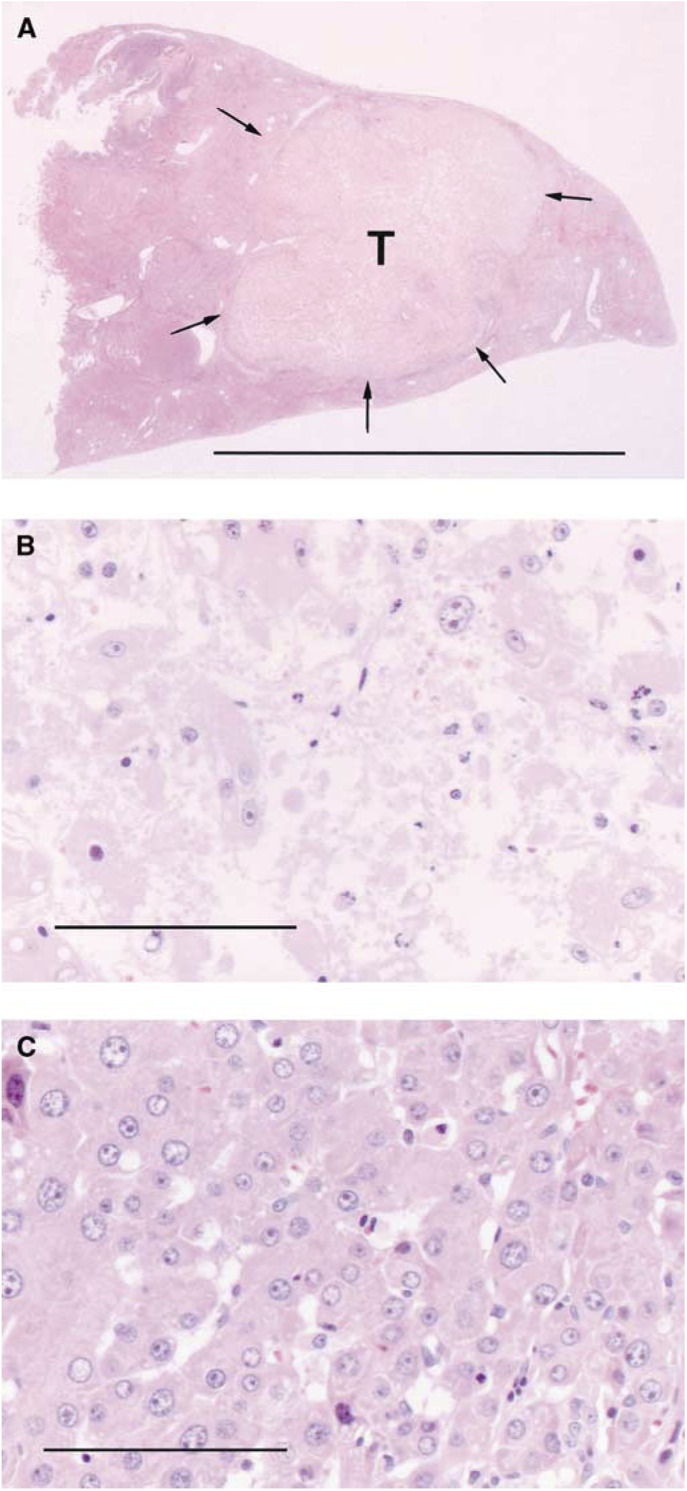
Histological sections of the liver including HCC treated with 30 J per tumour 3 h after 500 mg kg^−1^ ALA administration. (**A**) Necrosis was evident in HCC (T), and the necrotic area was 9 mm in diameter and 8 mm in depth. Laser irradiation was performed from the topside. The arrows indicate the necrotic area. (**B**) Almost all the HCC is occupied with necrotic tissue. (**C**) On the contrary, necrosis was not evident in the nontumoral tissue (FAH) surrounding HCC. Scale: (**A**) HE, a bar represents 10 mm. (**B**,**C**) HE, a bar represents 0.1 mm.

Based on the above, PDT was performed on HCC at 3 h after 500 mg kg^−1^ ALA administration before 30 J per tumour laser irradiation. In all treated tumours, necrosis was evident at 24 h after ALA-PDT. A representative case with the largest tumour is shown in Figure 7

. In this case, the tumour size was 9 mm in diameter, and therefore the total light dose was 47 J cm^−2^. HCC was completely necrotic; contrastingly, the nontumour tissue surrounding HCC was mostly viable. This indicates that HCC was selectively damaged after ALA-PDT.

## DISCUSSION

Recent advances in diagnostic imaging techniques have increased the likelihood of detecting HCC. Surgical resection is considered to be the most curative treatment for HCC. However, surgical resection is not possible in all patients with HCC, in particular those who have poor liver function due to underlying cirrhosis. Furthermore, the cancer frequently recurs even after curative surgical resection. Therefore, to improve the survival of patients with HCC, it is imperative to develop a new treatment modality which is nonsurgical and yet as effective as surgical resection. Although TAE, PEIT, and PMCT have been widely carried out as nonsurgical local treatments, the complications and long-term outcome after these treatments remain unsatisfactory. Recently, PDT has been successfully used as a local treatment in the management of patients with various malignant tumours. However, PDT has not been established for HCC yet. The aim of the present study was to investigate whether PDT, a therapy with high tumour selectivity, can be applied to HCC.

In this study, ALA was adopted as a photosensitiser because there had been some reports on the application of ALA to hepatoma. Van Hillegersberg *et al* (1992) have reported selective accumulation of porphyrin in hepatoma by oral administration of ALA in rat models in which colon cancer cells had been transplanted to the liver. In a subcutaneous transplant model of Morris hepatoma cells to the rat flank, Egger *et al* (1997) have reported the tumour selective accumulation of porphyrin after intraperitoneal administration of ALA. Moreover, diethylnitrosamine-treated rats were used as an HCC model for this study because this experimental model reflects the circumstances of human HCC better than subcutaneously implanted HCC or malignant tumour transplanted to the liver. This is the first demonstration of selective accumulation of PpIX in HCC after ALA administration and a large antitumour effect after ALA-PDT.

We indicated that PpIX after ALA administration accumulated predominantly in the HCC as compared with the nontumoral tissue of the liver in a chemically induced HCC model. First, the PpIX concentration correlated with fluorescence intensity (Figure 1) reached a peak value both in HCC and nontumoral tissues at 3 h after administration (Figure 3). This result was consistent with the report by Egger *et al* (1997) that PpIX accumulation reached its highest concentration at 4 h after ALA administration both in the liver and subcutaneously transplanted HCC. In addition, the fluorescence intensity of PpIX was higher in HCC than in nontumoral tissue at any time. Moreover, this difference was significant at 1 h after ALA administration (*P*<0.01) (Figure 3). This result meant that the ALA uptake was faster in HCC than in nontumoral tissue and progressively accumulated the porphyrin in the liver. At 3 and 6 h, PpIX concentration in the liver varied among the individual animals. A possible cause may have been differences in the amounts of ALA uptake to the liver because of individual differences in tumour size and number. Another cause may have been the instability of ALA in the near-neutral solution. To reduce side effects (Edwards *et al*, 1984), ALA was adjusted to pH 5.0–6.5. At pH 7.0, ALA became dimeric to the extent of 50% after 3 h (Elfsson *et al*, 1998), and the possibility cannot be denied that the efficacy of ALA rapidly deteriorated as pH approached neutrality.

Next, we investigated the ratio of PpIX concentration in HCC to that in nontumoral tissue in each individual because differences in PpIX accumulation in different tissue types in the same liver were important when photodynamic diagnosis (PDD) and PDT were performed. As shown in Figure 4, the mean of the ratio of the concentration in HCC to that in nontumoral tissue was significant at 1 h (*P*<0.001) and 3 h (*P*<0.05). At 6 h, there were thought to be individual differences in PpIX metabolism, causing scattered values. These suggest that PDD and PDT for HCC should be performed at 3 h after ALA administration.

The fluorescence macroscopic findings (Figure 5A) suggested that the detection of enhanced fluorescence of PpIX in the liver after ALA administration might be a potential diagnostic imaging modality for HCC. It may be useful in laparoscopic diagnosis of tumours near the surface of the liver and intraoperative investigation of residual tumour at the resected margin. Under the fluorescence microscope, fluorescence due to PpIX was seen homogeneously down to the deep portion of HCC, suggesting that PDT with ALA for HCC may be performed efficiently.

Finally, we demonstrated a large antitumour effect with ALA-PDT. PDT with an excimer dye laser 3 h after photosensitisation caused necrosis to a depth of 8 mm in the same experimental HCC model, and this necrosis was selective to HCC (Figure 7). The antitumour effect after ALA-PDT has been considered superficial because in many previous studies, ALA-PDT was performed after topical application and necrosis was reported to be shallow (Iinuma *et al*, 1995; Chang *et al*, 1996). However, Peng *et al* (1995) reported uniform distribution of PpIX throughout clinical noduloulcerative basal cell carcinoma after systemic administration of ALA, which was similar to our results (Figure 6A). The large antitumour effect in our HCC model due to this homogeneous distribution was comparable to the antitumour effect reported by Regula *et al* (1994) by ALA-PDT in cancer transplanted to the pancreas. These results suggest that optimal outcome in ALA-PDT for nodular lesions will be obtained by systemic administration.

Another possible reason for the large antitumour effect in this study is the relatively large dose of ALA compared with previous reports. Moreover, we cannot rule out the possibility of tissue specificity to the liver, because haem synthesis, of which ALA is a precursor, is very pronounced in the liver, and the metabolism may be even further enhanced in hepatoma, being generated in the liver. In *in vitro* experiments using hepatoma cells and normal liver cells, Kondo *et al* (1993) have reported high porphobilinogen deaminase activity and low ferrochelatase activity in hepatoma cells after ALA administration. Surface illumination, as used in our study, can produce a selective antitumour effect on large tumours. However, it is not certain that the laser dose and the manner of illumination used in this study are optimal. Further study is necessary to demonstrate the optimal conditions for ALA-PDT on HCC.

In order to apply ALA-PDT to human HCC, deeper penetration of the laser beam is required. To achieve this, we believe that interstitial irradiation is the most effective technique. A similar technique, PMCT, is being used clinically for the treatment of hepatoma, showing that technically it is not impossible (Seki *et al*, 1994). There have also been reports that interstitial irradiation was effective in treating transplanted hepatoma (Rovers *et al*, 1998). We expect that through further research ALA-PDT will become a safer and more effective treatment modality for HCC.
